# Advanced glycation end products promote VEGF expression and thus choroidal neovascularization via Cyr61-PI3K/AKT signaling pathway

**DOI:** 10.1038/s41598-017-14015-6

**Published:** 2017-11-02

**Authors:** Lijuan Sun, Tonglie Huang, Wenqin Xu, Jiaxing Sun, Yang Lv, Yusheng Wang

**Affiliations:** 1 Eye Institute of Chinese PLA and Department of Ophthalmology, Xijing Hospital, Fourth Military Medical University, Xi’an, 710032 China; 2 State Key Laboratory of Cancer Biology, Department of Biopharmaceutics, School of Pharmacy, Fourth Military Medical University, Xi’an, 710032 China

## Abstract

Choroidal neovascularisation (CNV) causes severe vision loss among old patients, especially those with diabetes. Previously, Cyr61 has been found to play a critical role in the pathogenesis of both AMD and diabetes. In the present study, we found that increased CNV severity together with higher expression of Cyr61 and VEGF in diabetes mice compared with control mice. Moreover, knockdown of Cyr61 decreased CNV severity. *In vitro* mechanism study revealed that the advanced glycation end products (AGEs) significantly increased the expression of Cyr61 in retinal pigment epithelial (RPE) cells, mimicking the effects of diabetes. In turn, the increased Cyr61 enhanced VEGF expression through FAK and PI3K/Akt pathways. Chemically blocking the above pathway significantly inhibited CNV formation, providing a new strategy for clinical prevention and treatment of CNV in related diseases.

## Introduction

CNV is now known to occur as a final common symptom in nearly 40 ophthalmic diseases, such as age-related macular degeneration (AMD), ocular histoplasmosis, myopic macular degeneration, idiopathic CNV and ocular trauma^1,2^. Currently, exploring the detailed mechanism of CNV and the clinical control strategy has become one of hotspots and difficulties in the research field of ophthalmology.

At present, it has been confirmed that age^3^, smoking^4^, genetics^5^ and cardiovascular diseases, are known risk factors for CNV^6^. Correlation research between diabetes and CNV progress has attracted more and more attention, suggesting roles of diabetes in CNV^7,8^. Though there is a lot of clinical epidemiological investigation at present, the molecular mechanism that diabetes affect CNV occurrence remains unclear because of a lack of basic experimental evidence supporting. Our previous study found that hyperglycemia played an important role in the development and progression of CNV^9^. Hyperglycemia promoted CNV possibly via recruitment of bone marrow-derived mesenchymal stem cell and induction of VEGF secretion by RPE cell^10^.

Cyr61, a very important cell matrix adjustment factor, has been found to play an important role in regulating cell adhesion^11^, migration^12^, proliferation^13^, angiogenesis^14^, inflammation^15^ and tissue reconstruction^16^. In our transcriptomics study, Cyr61 was identified as one of the most significantly differential genes in choroid tissue of diabetic mice (data not shown). However whether Cyr61 involved in CNV formation and the detailed molecular mechanism remain unknown.

In this study, we found for the first time that AGEs from the diabetes could increase the expression of Cyr61, which in turn activate the integrin-PI3K/AKT signal pathway and promote VEGF expression in RF/6 A cell. Simultaneously, MMPs expression were also found changed by Cyr61. Inhibiting AGEs formation or specific blocking Cyr61 signal pathway significantly restrain RF/6 A cell proliferation, migration and tube cavity formation, thus alleviate CNV severity caused by diabetes. Taken together, our study revealed an important role of AGEs-Cyr61-VEGF in CNV formation, and blocking the above pathway holds promising for CNV prevention and therapy.

## Results

### Hyperglycaemia upregulates Cyr61 and VEGF expression in laser-induced CNV mouse model

**Figure 1:**
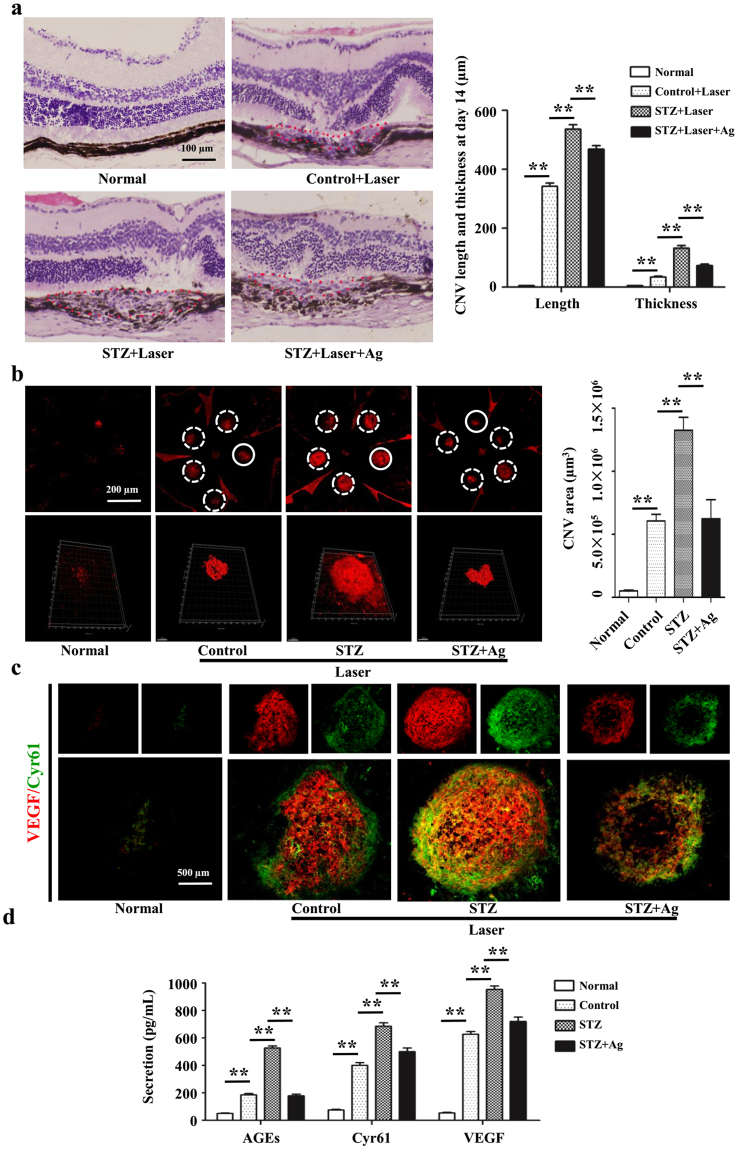
Hyperglycaemia upregulates Cyr61 and VEGF expression in laser-induced CNV. (**a**) CNV thickness and length of three groups in cross sections represent as indicated. The margins of CNV lesion are outlined by red dotted lines. Scale bar, 50 mm. (**b**) CNV areas of three groups in choroidal flatmounts represent as indicated. Blood vessels are stained by rhodamine-conjugated agglutinin (red) in choroidal flat mounts. Volume of CNV was calculated by 3D reconstruction software. (**c**) The expression patterns of Cyr61 and VEGF at CNV sites were performed by Immunofluorescence staining. (**d**) Statistical analysis of the AGEs, Cyr61 and VEGF ELISA data. Ag, Amino guanidine. Values are the mean ± SEM of at least 3 independent experiments. **P < 0.01 compared between the indicated groups.

**Figure 2:**
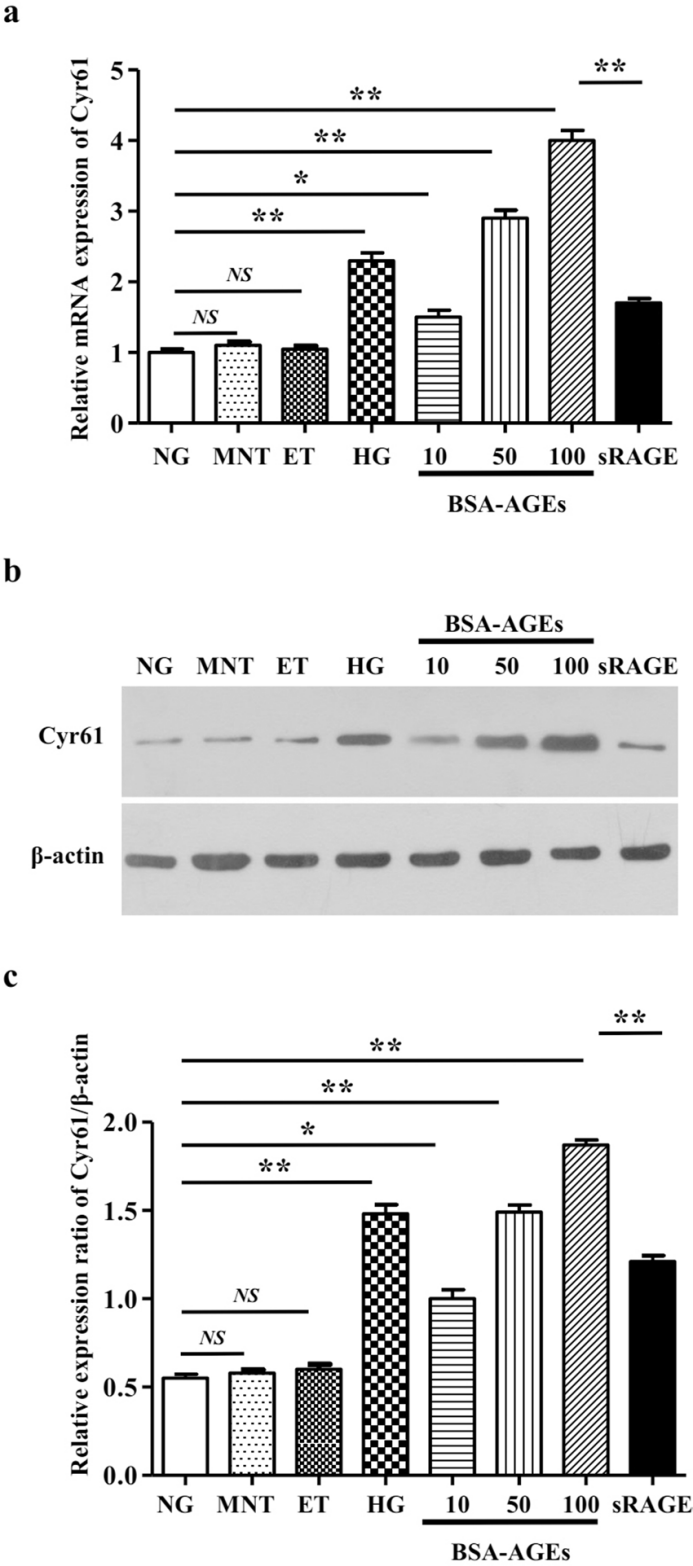
AGEs-RAGE interaction induces Cyr61. The expression level of Cyr61 in ARPE-19 cell with BSA-AGEs stimulation was performed by qPCR (**a**) and western blot (**b**). (**c**) Quantification data of Fig. 2b.

**Figure 3:**
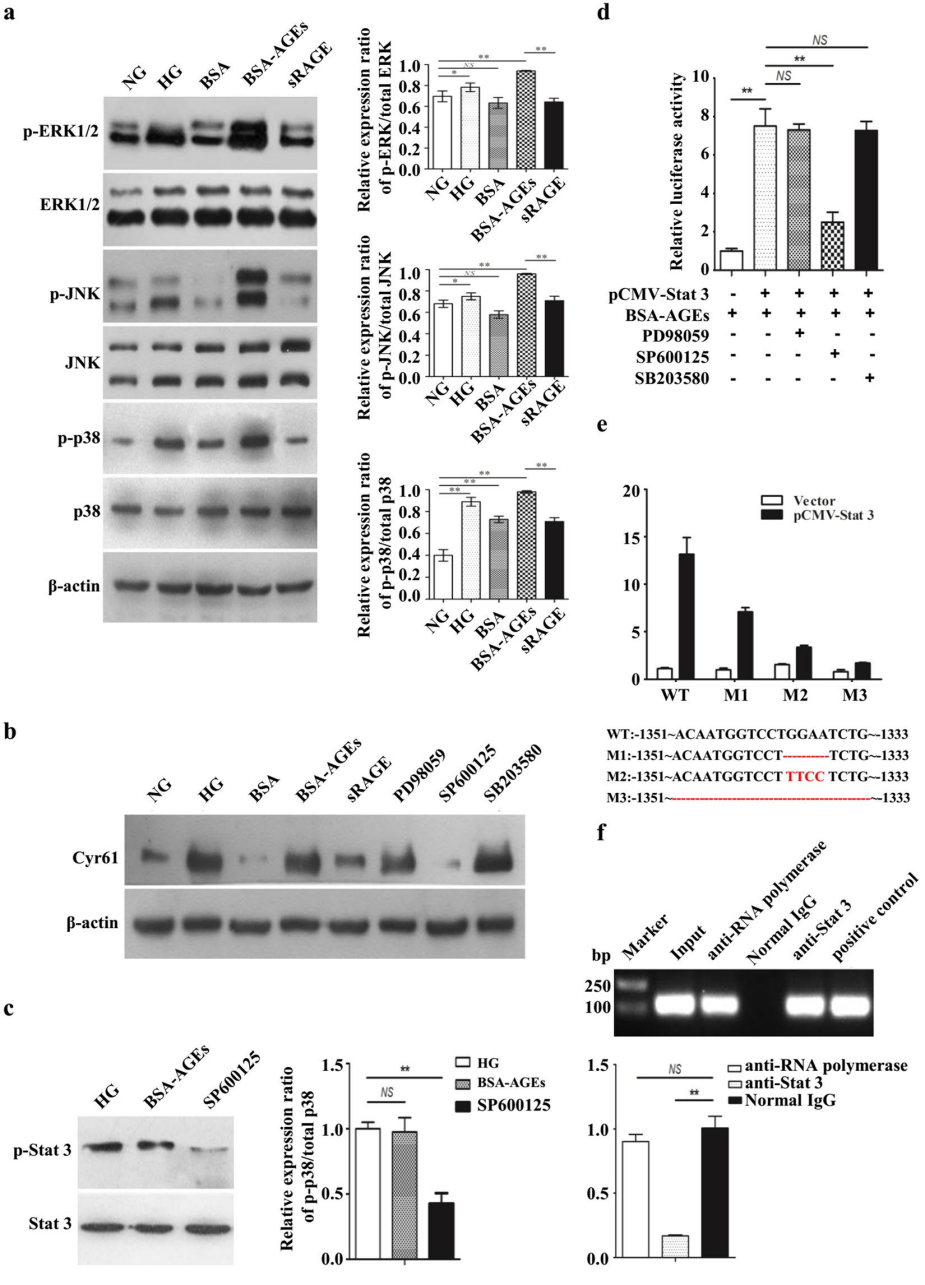
AGEs-RAGE interaction induces Cyr61 via activation of Stat 3. (**a**) MAPK protein expression levels were measured by western blot analysis. Protein expression was normalized to β-actin. (**b**) The protein expression level of Cyr61 in ARPE-19 cell with MAPK inhibitors stimulation was determined by western blot. (**c**) The protein expression level of Stat 3 in ARPE-19 cell with JNK inhibitors stimulation was determined by western blot. (**d**) Stat 3 accelerates Cyr61 promoter activity as evaluated by a dual-luciferase reporter assay. (**e**) Deletion of the Stat 3-binding sites in Cyr61 promoter prevents Stat 3-mediated transcriptional activation. (**f**) Stat 3 DNA binding activity was examined by ChIP. NG, normal glucose. HG, high glucose. Values are the mean ± SEM of at least 3 independent experiments. NSP > 0.05, *P < 0.05 and **P < 0.01 compared between the indicated groups.

Streptozotocin was used to induce diabetic mouse model. Compared with control group, there were obvious RPE cells appeared and Bruch’s membrane rupture, more cell proliferation and migration, and increased new blood vessels in the diabetic mice treated with laser (Fig. 1a). Accordingly, Cyr61 and VEGF were high expression in CNV area (Fig. 1c). Since AGEs are key mediators of diabetes, aminoguanidine, which could suppress AGEs formation, was used to confirm the role of AGEs in CNV. As expected, aminoguanidine treatment could ameliorate CNV severity (Fig. 1a and b). In addition, aminoguanidine treatment also reduced the production of Cyr61 and VEGF (Fig. 1c,d). We next explored the mechanism how diabetes and AGEs activates Cyr61. No obvious endotoxic effects were observed by the commercially obtained BSA-AGEs. As shown in Fig. 2a–c, both the mRNA and protein levels of Cyr61 increased when ARPE-19 cell was stimulated by hyperglycemia and AGEs, while no effects were observed by high mannitol. And this effect can be antagonized by sRAGE. We then found that MAPK activity could be induced by AGEs (Fig. 3a). However, only JNK inhibitor SP600125 could block the increased Cyr61 induced by AGEs (Fig. 3b). These data indicated that AGEs could interact with RAGE receptor on RPE cell surface and activate JNK signaling pathways. Through bioinformatics analysis, we detected that the binding site of transcription factor Stat 3 involved in Cyr61 promoter region. Our previous work also brought to light that hyperglycaemia promotes the development of CNV by activating Stat 3 signaling in RPE cells^9^. Therefore, we examined whether AGEs promoted Stat 3 activation through JNK signaling pathway. The result showed that AGEs could induce transcription factor Stat 3 activation, which can be blocked by JNK inhibitor (Fig. 3c). We confirmed that the activation of Stat 3 mediated by AGEs could significantly enhance the transcriptional activity of truncation in Cyr61 promoter region (Fig. 3d). Mutation of the Stat 3 binding site at −1351 bp to −1333 bp of the Cyr61 promoter resulted in reduced basal transcription (Fig. 3e). Thus, the Stat 3 site at −1351 bp to −1333 bp of the Cyr61 promoter is critical for basal Cyr61 transcription in RPE cell. To determine if Stat 3 directly targets the Cyr61 promoter, ChIP analysis was performed. An anti-Stat 3 antibody pulled down higher amounts of Cyr61 promoter DNA than the IgG control based on the results of quantitative analysis (Fig. 3f).

### Cyr61-FAK-PI3K signaling boosts the production of VEGF

**Figure 4:**
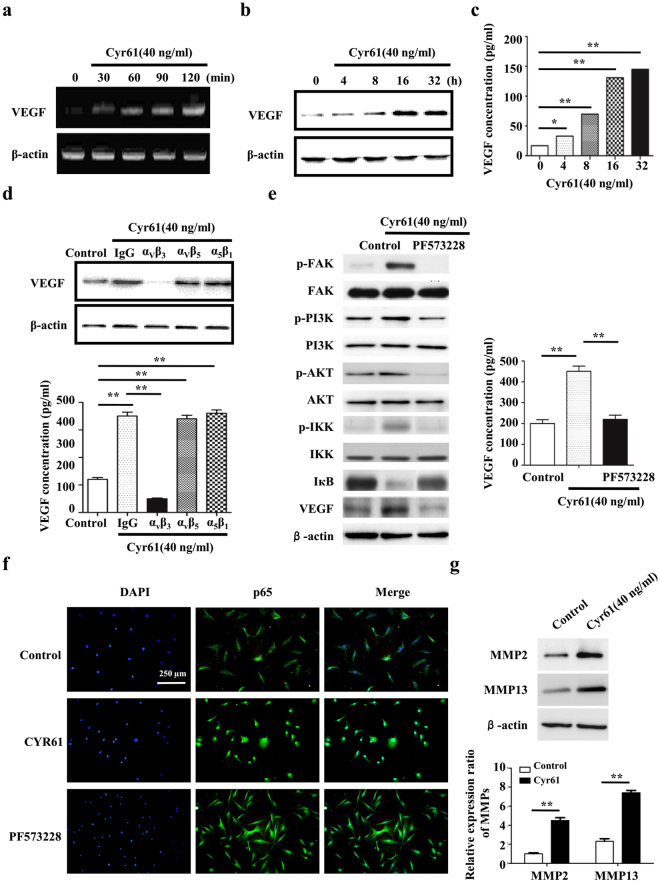
Cyr61-FAK-PI3K signaling boosts VEGF expression. The effect of Cyr61 on the expression level of VEGF in RF/6 A cell was performed by RT-PCR (**a**), western blot (**b**) and ELISA (**c**). (**d**) RF/6 A cells were treated with an anti-α_ν_β_3_, anti-α_ν_β_5_, or anti-α_5_β_1_ blocking antibody for 1 h. Following incubation with the inhibitors, the cells were stimulated with Cyr61 (40 ng/mL), and the levels of total (top panel) and secreted (bottom panel) VEGF protein were measured through western blot analysis and ELISA, respectively. (**e**) The cells were pretreated with a FAK inhibitor (PF573228) for 1 h, then stimulated with Cyr61 (40 ng/mL). The total (left panel) and secreted (right panel) MCP-1 protein levels were measured through western blot and ELISA analyses, respectively. (**f**) RF/6 A cells were stimulated as indicated and an immunofluorescence assay was performed 24 h later to detect the activation and translocation of NF-κB p65. (**g**) The effect of Cyr61 on the expression level of MMPs in RF/6 A cell was performed by western blot. Values are the mean ± SEM of at least 3 independent experiments. *P < 0.05 and **P < 0.01 compared between the indicated groups.

Since the essential role of VEGF in angiogenesis, we next investigate the effect of Cyr61 on VEGF via RT-PCR, western blot and ELISA. As shown in Fig. 4a,b and c, VEGF expression was increased by exogenous Cyr61. Because Cyr61 acts through binding to different integrin receptors^17^, anti-ανβ3, anti-ανβ5, and anti-α5β1 blocking antibodies were used respectively. The results showed that Cyr61 regulated the expression of VEGF could be blocked by anti-α_ν_β_3_ (Fig. 4d).

The phosphoinositide 3-kinase (PI3K)/Akt signaling pathway is essential to angiogenesis. In addition, a previous study indicated that the PI3K/Akt pathway is required for the hypoxia-induced expression of HIF-1α and VEGF in choroidal neovascularization^18^. We thus examined the effects of Cyr61 on the phosphorylation of PI3K, Akt and IKK over time. Pretreatment with the FAK inhibitor (PF573228) inhibited the phosphorylation of PI3K, Akt and IKK as well as the expression of VEGF (Fig. 4e). Moreover, upregulation of Cyr61 accelerated NF-κB activation and translocation, whereas FAK inhibitor blocked the activation of NF-κB (Fig. 4f). These results indicate that the FAK and PI3K/Akt pathways play a role in Cyr61-mediated VEGF expression, mainly via NF-κB activation. Furthermore, we demonstrated that Cyr61 could promote the expression of MMP2 and MMP13 (Fig. 4g).

### Cyr61 Promotes RF/6 A cell proliferation, migration and tube formation

**Figure 5:**
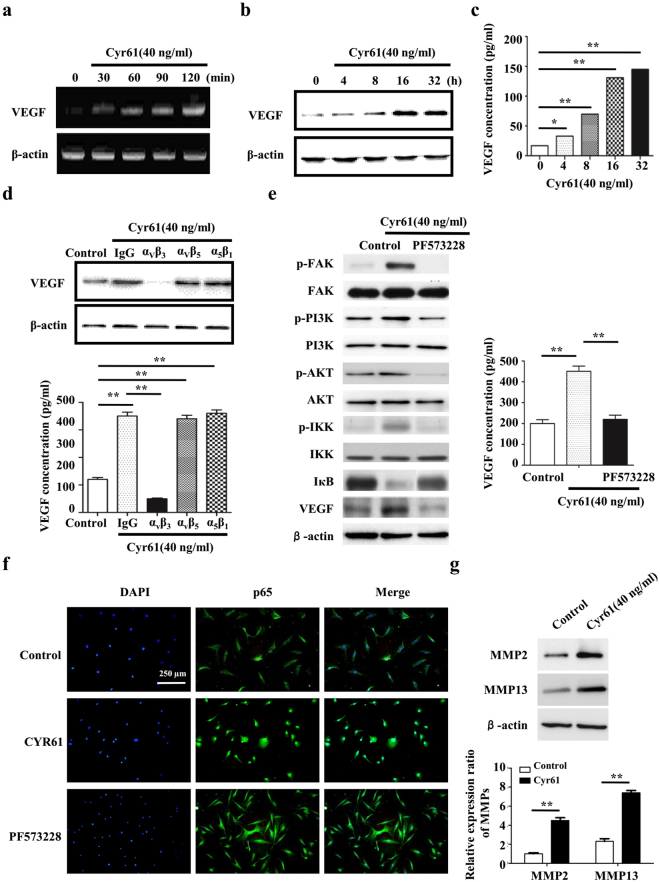
Cyr61 promotes cell proliferation, migration and tube formation. (**a**) The effect of Cyr61 on the proliferation of RF/6A was evaluated by MTT assay. (**b**) The effect of Cyr61 on the migration of RF/6A was performed by Transwell assay. (**c**) The effect of Cyr61 on the tube formation of RF/6A was performed by *in vitro* tube formation assay. Values are the mean ± SEM of at least 3 independent experiments. **P < 0.01 compared between the indicated groups.

To further verify the functional effects of Cyr61 on RPE cells, cell proliferation, migration and angiogenesis assays were carried out. As shown in Fig. 5a,b and c, inhibiting the formation of AGEs or gene silencing of Cyr61 could markedly repress RF/6 A cell proliferation, migration and tube formation.

### Cyr61 interference *in vivo* alleviates the leakage area of CNV

**Figure 6:**
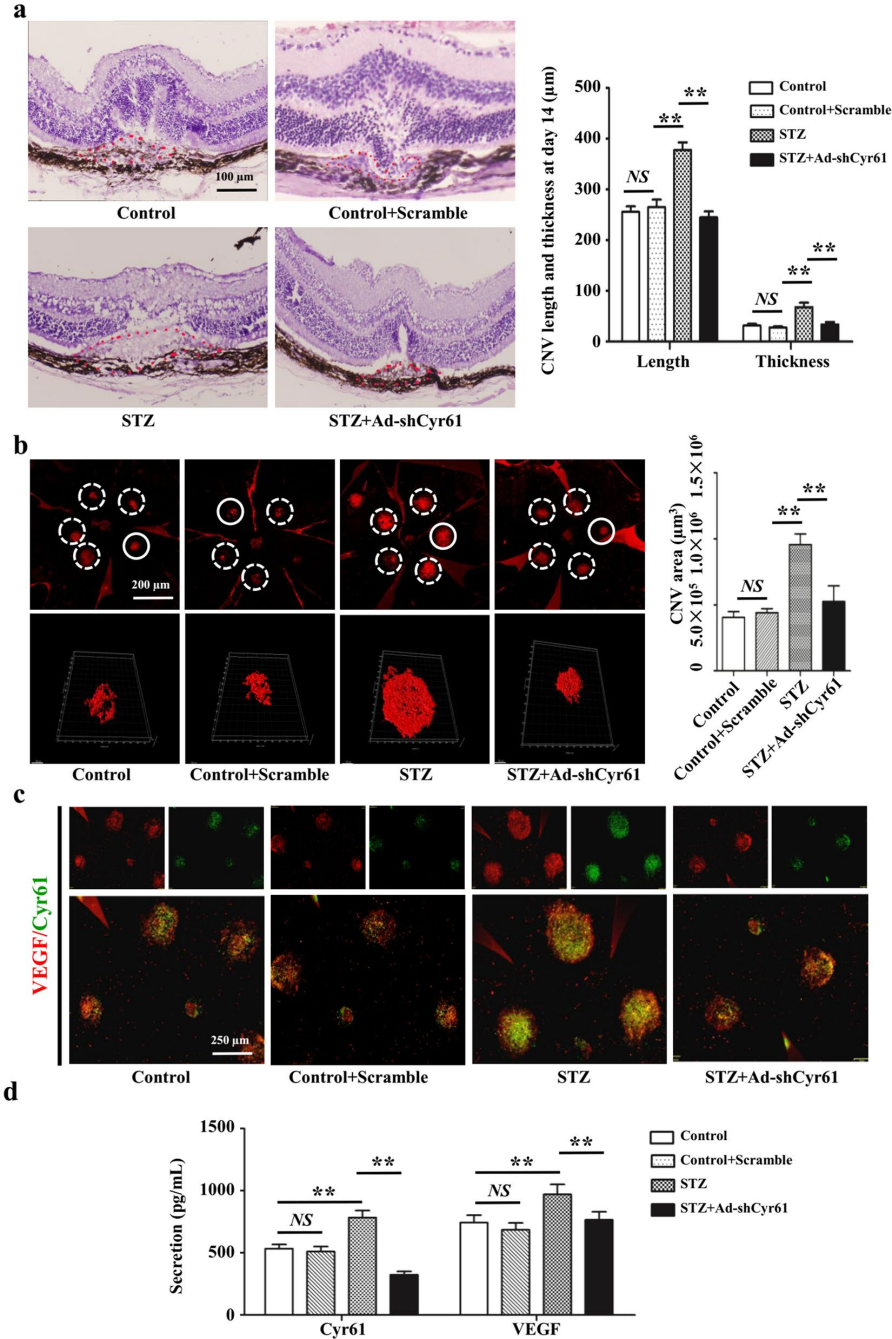
Cyr61 knockdown suppresses VEGF expression and alleviates the leakage area of CNV. (**a**) CNV thickness and length of three groups in cross sections represent as indicated. The margins of CNV lesion are outlined by red dotted lines. Scale bar, 50 mm. (**b**) CNV areas of three groups in choroidal flatmounts represent as indicated. Blood vessels are stained by rhodamine-conjugated agglutinin (red) in choroidal flat mounts. Volume of CNV were calculated by 3D reconstruction software. (**c**) The expression patterns of Cyr61 and VEGF at CNV sites were performed by Immunofluorescence staining. (**d**) Statistical analysis of the Cyr61 and VEGF ELISA data. Values are the mean ± SEM of at least 3 independent experiments. **P < 0.01 compared between the indicated groups.

All of the above results suggest that knockdown of Cyr61 could be beneficial for CNV therapy. Significant knockdown effects were observed by intravitreal injection with shCyr61. Knockdown of Cyr61 had minimal effects on diabetes itself, as no blood glucose and body weight change were observed (Data not shown). However, intravitreal injection with shCyr61 could significantly reduce the CNV severity of the diabetes mice, as seen from the choroidal flatmout and HE staining (Fig. 6a and b). Moreover, we also demonstrated that specific interfering Cyr61 in CNV area could decrease local VEGF expression and secretion (Fig. 6c and d).

## Discussion

CNV, a process involving the growth of new blood vessels from the choriocapillaris across the retinal pigment epithelium (RPE) layer and Bruch’s membrane and their extension into the subretinal space, is a major feature of several ocular diseases that cause vision loss, such as wet age-related macular degeneration (AMD), ocular histoplasmosis syndrome, and pathological myopia^19,20^. The etiology of CNV is multifactorial and complicated, and a number of genetic and environmental factors have been cited as possible risk factors for its formation^21^.

Our previous study found that hyperglycemia plays an important role on the development and progression of CNV, while the detailed mechanism remains largely unknown.

The current study demonstrated that Cyr61 and VEGF are overexpressed in diabetic mice. Correlation analyses revealed a significant positive correlation between the expression levels of Cyr61 and AGEs. Cyr61 is a secreted matricellular protein that associates with the cell surface and the extracellular matrix via binding to integrins or heparan sulfate proteoglycans^12^. Functionally, Cyr61 mediates cell proliferation, adhesion, migration, and differentiation and promotes angiogenesis^22^. Cyr61 has been implicated in a myriad of disease processes^23,24^, including age-related macular degeneration^25,26^. Although the specific role of Cyr61 in choroidal neovascularization is not well understood, evidence for its participation in eye diseases is increasing. Zhang *et al*. reported that Cyr61 are likely to be involved in the pathogenesis of diabetic retinopathy, and may play a role in the course of neovasculation^27^. Di *et al*. suggest that Cyr61 plays an important role in RNV in ROP, and may thus be a potential target for the prevention and treatment of ROP^28,29^. You *et al*. demonstrated that hypoxia controlled the transcriptional regulation of the Cyr61 gene in RF/6 A cells by cooperation of HIF-1a and c-Jun/AP-1. Cyr61 might play an important role in ischemic retinal diseases, such as PDR^30^. They subsequently proved that Cyr61 not only takes part in ocular angiogenesis but also induces MCP-1 expression in the retina^31^. Our study here found that AGEs promoted Stat 3 activation through JNK signaling pathway, which in turn bind to the Cyr61 promoter and drive CYR61 promoter activation.

Several biological pathways have been implicated in the pathogenesis of age-related macular degeneration^32^. These include senescence, shown by lipofuscin accumulation in retinal pigment epithelium cells, choroidal ischaemia, and oxidative damage^33^. More recently, attention has been focused on the function of VEGF in light of its role as a therapeutic target^34^. VEGF is a key regulator of angiogenesis, and withdrawal or interference with its function leads to cessation of vascular growth and neovascular regression^35^. VEGF expression has been shown in experimental choroidal neovascularisation, and shown to induce choroidal neovascularisation growth in animals. In the present study, we demonstrated that the induction of VEGF expression in RF/6 A cell by AGEs is due at least in part to Cyr61 abundance. Moreover, we demonstrated that the FAK and PI3K/Akt pathways play a role in Cyr61-mediated VEGF expression. Knockdown of Cyr61 by adenovirus significantly reduce the CNV severity of the diabetes mice.

**Figure 7:**
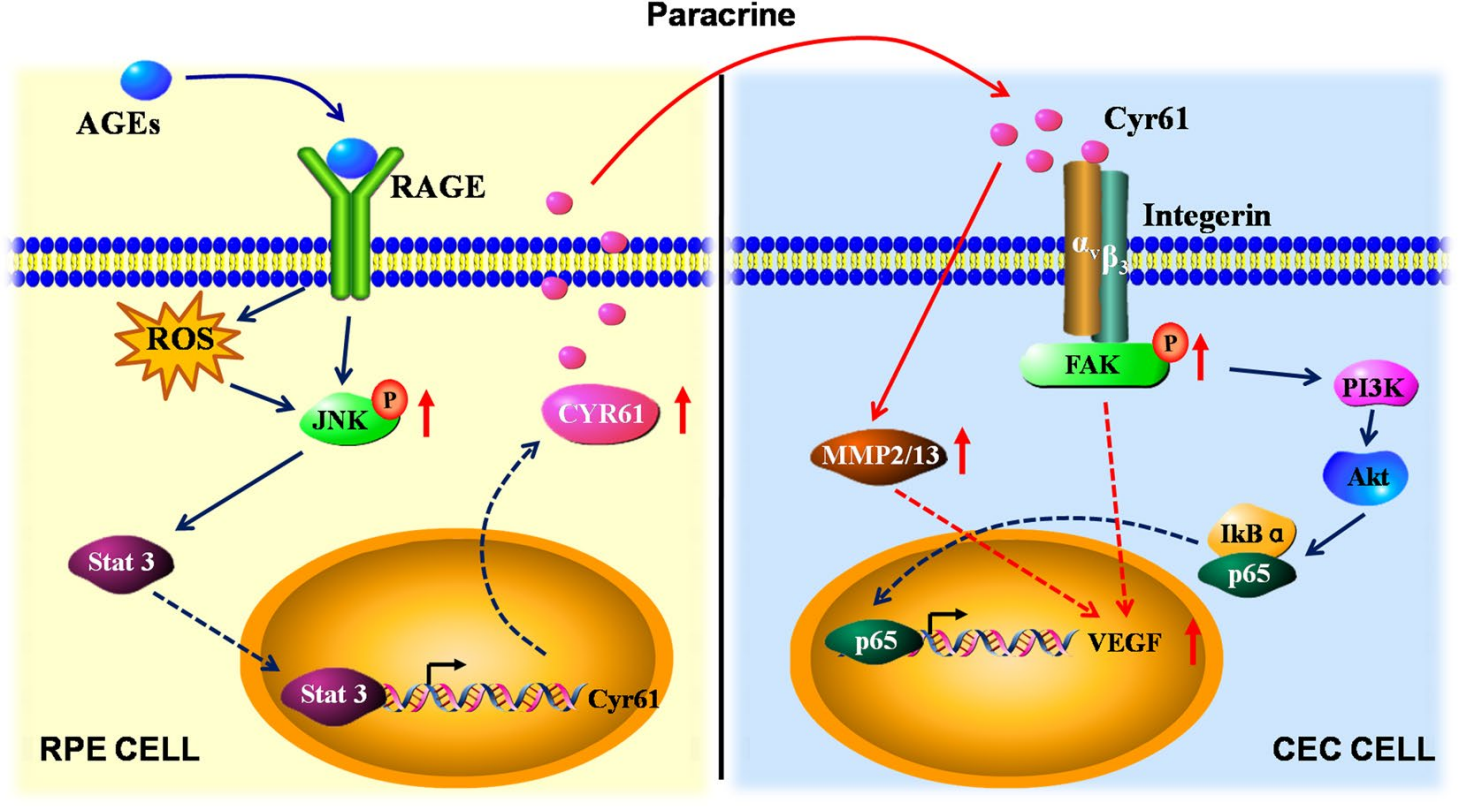
Schematic model of the AGEs-Cyr61-VEGF pathway observed in this study. AGEs binds to RAGE on ARPE-19 cell and induces the activation of JNK. Then, activated JNK enhances Cyr61 expression via activation of the Stat 3. Next, the increased Cyr61 induces VEGF secretion modulated by PI3K-Akt signaling pathway. Finally, angiogenic factor (e.g. VEGF, MMPs) stimulation induces choroidal neovascularization. However, how the induced CYR61 acts on RF/6A cell regulates MMPs expression remains to be determined. ROS, reactive oxygen species; JNK, reactive oxygen species; FAK, focal adhesion kinase.

In summary, we found for the first time that AGEs could interact with RAGE receptor, and regulate the expression of Cyr61. Cyr61 in turn could activate the intergrin-PI3K/AKT signal pathway, accelerate NF-κB nuclear translocation and promote VEGF and MMPs expression in RF/6 A cell. Inhibiting AGEs formation or specific blocking Cyr61 could significantly restrain RF/6 A cell proliferation, migration and tube cavity formation, thus alleviating CNV severity caused by diabetes (Fig. 7). Taken together, our data indicate AGEs-Cyr61-VEGF signal pathway involved in the regulation of CNV formation process, and chemically blocking the above pathway significantly inhibited CNV formation, providing a new strategy for clinical prevention and treatment of CNV in related diseases.

## Methods

### Ethic statements

The animal studies were carried out in strict accordance with the Detailed Rules for the Administration of Animal Experiments for Medical Research Purposes issued by the Ministry of Health of China, and were approved by the Animal Experiment Administration Committee of The Fourth Military Medical University. All efforts were made to keep pain and suffering to a minimum.

### Cell culture

Human RPE cell line ARPE-19 was purchased from the American Type Culture Collection (Manassas, VA and Rockville, MD, USA). Monkey chorioretinal vessel endothelial cell (RF/6 A) was obtained from the Cell Bank of the Chinese Academy of Sciences (Shanghai, China) and was identified as being of endothelial origin with cellular morphology, growth pattern, ultrastructure, immunocytochemistry, and immunodiffusion^36^. All cells were grown in Dulbecco’s modified Eagle’s medium (DMEM) supplemented with 10% fetal bovine serum (FBS), 100 μg/mL streptomycin, and 100 U/mL penicillin and maintained at 37 °C in a humidified environment with 5% CO_2_. The integrin pathways were blocked by adding the neutralizing antibodies. Mouse anti-α_ν_β_3_, anti-α_ν_β_5_, and anti-α_5_β_1_ blocking antibodies were purchased from Millipore (Billerica, MA, USA).

### Mouse model of CNV

Streptozotocin (STZ) (Sigma, Darmstadt, Germany) was used to produce experimental diabetes. Briefly, C57/BL6 mice were injected intraperitoneally with a low dose of STZ (50 mg/day/kg body weight, dissolved in fresh cold citrate buffer [pH 4.5]) for 5 consecutive days. Hyperglycemia (glucose more than 22 mmol/L) was confirmed 2 weeks later using a reflectance meter.

CNV was generated by laser photocoagulation-induced rupture of Bruch’s membrane as described previously^37^. Briefly, the mice were anesthetized, and their pupils were dilated. Laser photocoagulation with a wavelength of 532 nm, a spot size of 75 mm, a duration of 0.1 second, and an intensity of 90 mW was delivered using a slit lamp and a corneal contact lens. The burns were performed at positions that were 1.5–2 disc diameters away from the optic nerve. Only those laser spots at which the rupture of Bruch’s membrane was confirmed via the presence of a vaporisation bubble and the absence of haemorrhaging were considered successful.

### Evaluation of CNV severity

The size of CNV was evaluated 14 days after laser photocoagulation by choroidal flat-mount and histopathological examination. Choroidal flat mounts were prepared after CNV induction (6 spots per eye) in accordance with a previously described protocol^38^. Anesthetised mice were transcardially perfused with a 0.9% saline solution followed by a 4% paraformaldehyde solution. The entire ocular globes were enucleated, and the anterior segment and neural retina were removed from each globe. The remaining RPE-choroid-sclera complex was flatmounted using six or more radial cuts, after which the flatmount preparations were permeabilised in a 0.2% Triton X-100 solution for a period of 24 h prior to transferring them to a 1:1000 solution of rhodamine-conjugated Ricinus communis agglutinin (Vector Laboratories, Burlingame, CA, USA). Choroidal preparations were incubated with the agglutinin for 24 h and were subsequently washed in a 0.01 M Tris-Buffered Saline Tween-20 (TBST) solution for another 24 h. Flatmounts were subsequently examined and photographed using confocal laser scanning microscopy (Olympus Corporation, Tokyo, Japan) and the CNV area of each preparation was assessed using the image pro plus software program (IPP 6.0). Individual lesions with surface areas of more than 0.50 disc areas (DAs) were defined as having CNV. Histopathological analysis was performed according to a previously described procedure^39^. Mice that were examined using light microscopy were killed on the 14th day after photocoagulation (6 spots per eye) and their eyes were enucleated. Eyecup preparations were fixed via incubation in Bouin’s fixative (Zhongshan Biotechnology Company, Beijing, China) at 4 uC for a period of 24 h. The fixed tissues were embedded in paraffin, serially sectioned into 3-mm slices and stained with hematoxylin.

### Enzyme-linked immunosorbent assay

The ocular levels of AGEs, Cyr61 and VEGF protein expression on day 3 after photocoagulation (10 spots per eye) were determined using a mouse ELISA kit (USCN Life Science and Technology, Wuhan, China). On the 3rd day after photocoagulation, the eyes were removed and prepared for ELISA according to a previously reported protocol^40^. The eyes were quick-frozen in 200 mL of phosphate-buffered saline solution (pH 7.4) that contained 0.05% phenylmethylsulfonyl fluoride and they were then manually homogenised on ice and exposed to three freeze-thaw cycles in liquid nitrogen and wet ice. The homogenates were centrifuged in a refrigerated desktop centrifuge to pellet any insoluble material, and the supernatants were collected. A human ELISA kit was used to measure the expression levels of VEGF protein that were secreted by human RPE cells under different concentration of Cyr61 (USCN Life Science and Technology, Wuhan, China) in accordance with the manufacturer’s instructions. Representative results were expressed in pg/ml.

### *In vitro* cell proliferation assay

To test the effect of ARPE-19 cells on RF/6 A cell proliferation with existence of AGEs, a proliferation assay model was used. The RF/6 A cells were seeded in 96-well plates at 3 × 10^3^ cells/well. Next, cells were treated with Cyr61 shRNA before 0.4 μm pore-size inserts were placed in the wells. Medium containing AGEs was added and was changed every 2 days. At the end of each time point, 20 μL of 5 mg/mL 3-(4,5-dimethylthiazol-2-yl)-2,5-diphenyl tetrazolium bromide (MTT; Sigma, St. Louis, MO) was added to each well for 4 h at 37 °C. The supernatants were then removed and the formazan dye was dissolved in DMSO. The absorbance was measured using a microplate reader (Molecular Device, Menlo Park, CA, USA) at a wavelength of 490 nm.

### *In vitro* cell migration assay

To test the effect of ARPE-19 cells on RF/6 A cell migration with existence of AGEs, a migration assay model was employed. The ARPE-19 cells were plated at a density of 1 × 10^5^ cells/cm^2^ in 24-well plates with medium containing AGEs added to the wells for 12 h. The RF/6 A cell migration assay was performed using Matrigel-coated, Costar Transwell inserts with 8.0 mm pore size. Briefly, 5 × 10^4^ RF/6 A cells were seeded on the inserts and incubated with DMEM containing 1% FBS. After 1 h for attachment, the inserts were transferred to 24-well plates as described above. After incubation for 4 h, the inserts were fixed with 4% paraformaldehyde, stained with 0.1% crystal violet for 30 min, and photographed under a light microscope (Olympus, Tokyo, Japan). Five random fields (×200) were chosen in each insert, and the cell number was quantified manually.

### *In vitro* tube formation assay

ARPE-19 cells were plated in the transwells with 8 μm pore size inserts, which were put in the wells where the RF/6 A cells had been plated. Briefly, matrigel was pipetted into pre-chilled 24-well plates (150 mL matrigel per well) and polymerized for 45 min at 37 °C. After gel polymerization, 4 × 10^4^ RF/6 A cells were seeded in each well and incubated with DMEM supplemented with 0.5% FBS for 24 h at 37 °C in humidified air with 5% CO_2_. The enclosed networks of tubes were photographed 12 h later using Cannon Power Shot A640 camera on Zeiss inverted microscope with magnification × 100. Five fields from each chamber were counted and averaged.

### Quantitative real-time PCR (qPCR)

Total RNA was extracted from cells using the Trizol reagent (TaKaRa Bio Inc., Japan) according to the manufacturer’s instructions. Complementary DNA was prepared by using a reverse transcription kit from TaKaRa. Real-time PCR was performed by using a kit (SYBR Premix EX Taq, TaKaRa) and the ABI PRISM 7300 real time PCR system. The primer sequences were listed in Supplementary Table S1. β-actin was used as an internal control.

### Western Blot Analysis

Cells were harvested and proteins were extracted using cell lysis buffer supplemented with 0.3% PMSF and proteinase and phosphatase inhibitors. Proteins were separated by SDS-PAGE and transferred to PVDF membrane (Millipore, Bedford, MA, USA). After blocking in 5% skim milk powder for 2 h at room temperature, and the samples were incubated overnight at 4 °C with primary antibodies including ERK1/2 (#9102), p-ERK1/2 (#9101), JNK1/2 (#9252), p-JNK (#9251), p38 (#9212), p-p38 (#9211), Stat 3 (#9139), p-Stat 3 (#4113), FAK (#3285), p-FAK (#8556), PI3K (#3011), p-PI3K (#13857), Akt (#4691), p-Akt (#4060), IKK (#3416), p-IKK (#8766), IκB (#4814) and which were obtained from Cell Signaling Technology (Beverly, MA, USA), VEGF (sc-7269), MMP2 (sc-10736), MMP13 (sc-12363) and β-actin (sc-47778), which were obtained from Santa Cruz Biotechnology (Santa Cruz, CA, USA). After washing three times with TBST, the samples were incubated 1 h at room temperature with a secondary antibody. Bound antibodies were detected using the chemiluminescent substrate ECL (Pierce, Rockford, IL).

### Dual-luciferase reporter assay

Luciferase reporter assays were performed to characterize the pGL3-Cyr61 promoter. The pRL-TK vector, which expresses *Renilla* luciferase, was cotransfected to correct for differences in both transfection and harvest efficiencies. Cell lysates were collected 48 h after transfection, and luciferase activity was measured using a Dual-Luciferase Reporter Assay System (Promega). The activity of the firefly luciferase reporter was normalized to that of the *Renilla* luciferase.

### Chromatin immunoprecipitation (ChIP) assay

A ChIP assay was performed using a commercially available kit (Millipore) according to the manufacturer’s instructions. Briefly, cells were crosslinked in 1% formaldehyde for 10 min at room temperature. Then, the anti-Stat 3 antibody and control IgG were used for the ChIP assay. The precipitated DNA fragments were quantified by PCR using the primer sets shown in Supplementary Table S1.

### Statistical analysis

All reported data are representative of at least three independent experiments. Data are expressed as the mean ± SD and were analyzed by the Student’s *t*-test. *P* < 0.05 was considered statistically significant.

## Electronic supplementary material

Supplementary information
