# Supplementary material for: Advanced glycation end products promote VEGF expression and thus choroidal neovascularization via Cyr61-PI3K/AKT signaling pathway
Source: Sci Rep. 2017 Nov 2;7:14925. doi: 10.1038/s41598-017-14015-6 (PMC5668426; doi:10.1038/s41598-017-14015-6)
Supplement: Supplementary file 1 — Supplementary information [file 41598_2017_14015_MOESM1_ESM.doc]

**Advanced glycation end products promote VEGF expression and thus choroidal neovascularization via Cyr61-PI3K/AKT signaling pathway**

**Lijuan Sun^1, +^, Tonglie Huang^2, +^, Wenqin Xu^1^, Jiaxing Sun^1^, Yang Lv^1^, and Yusheng Wang^1, *^**

^1^Eye Institute of Chinese PLA and Department of Ophthalmology, Xijing Hospital, Fourth Military Medical University, Xi'an 710032, China;

^2^State Key Laboratory of Cancer Biology, Department of Biopharmaceutics, School of Pharmacy, Fourth Military Medical University, Xi’an 710032, China

^*^Correspondence:

Yusheng Wang, Eye Institute of Chinese PLA and Department of Ophthalmology, Xijing Hospital, Fourth Military Medical University, Chang-Le Xi Street #169, Xi’an 710032, China, Tel: +86-29-84775371; Fax: +86-29-84775371; Email: wangys003@126.com.

**^+^** These authors contributed equally to this work.

# Supplementary information

**Table S1.** The nucleotide sequence of primers

| **Gene or products** | | **Primers** |
| --- | --- | --- |
| β-actin (NM_001101.3) | Forward: 5’-GCGTGACATTAAGGAGAAG-3’ | |
|  | Reverse: 5’-GAAGGAAGGCTGGAAGAG-3’ | |
| Cyr61(NM_010516.2) | Forward: 5’-GCTCTAGAATGAGCTCCCGCATCGCCA-3’ | |
|  | Reverse: 5’-GGAATTCTTAGTCCCTAAATTTGTGAATGTC-3’ | |
| VEGF(NM_001171623.1) | Forward: 5’-ATGGCAGAAGGAGGAGGG-3’ | |
|  | Reverse: 5’-CGAAACGCTGAGGGAGGCT-3’ | |
| ChIP | Forward: 5’-TGGGCAGGAGATGCTAAATAAGA-3’ | |
|  | Reverse: 5’-GCTCCTTGGTGCTAGGCAG-3’ | |
